# A comparative study of federated learning methods for COVID-19 detection

**DOI:** 10.1038/s41598-024-54323-2

**Published:** 2024-02-16

**Authors:** Erfan Darzi, Nanna M. Sijtsema, P. M. A. van Ooijen

**Affiliations:** 1 Harvard Medical school, Harvard University, 300 Longwood avenue, Boston, United States; 2 Department of Radiotherapy, University Medical Center Groningen, University of Groningen, Hanzeplein 1, Groningen, The Netherlands; 3 Machine Learning Lab, Data Science Center in Health (DASH), University Medical Groningen, University of Groningen, Hanzeplein 1, Groningen, The Netherlands

**Keywords:** Federated learning, Medical image analysis, COVID-19, Privacy preserving machine learning, Medical imaging, Medical research

## Abstract

Deep learning has proven to be highly effective in diagnosing COVID-19; however, its efficacy is contingent upon the availability of extensive data for model training. The data sharing among hospitals, which is crucial for training robust models, is often restricted by privacy regulations. Federated learning (FL) emerges as a solution by enabling model training across multiple hospitals while preserving data privacy. However, the deployment of FL can be resource-intensive, necessitating efficient utilization of computational and network resources. In this study, we evaluate the performance and resource efficiency of five FL algorithms in the context of COVID-19 detection using Convolutional Neural Networks (CNNs) in a decentralized setting. The evaluation involves varying the number of participating entities, the number of federated rounds, and the selection algorithms. Our findings indicate that the Cyclic Weight Transfer algorithm exhibits superior performance, particularly when the number of participating hospitals is limited. These insights hold practical implications for the deployment of FL algorithms in COVID-19 detection and broader medical image analysis.

## Introduction

Coronaviruses comprise a family of viruses known to cause respiratory and intestinal illnesses in both humans and animals. Among these viruses, the variants responsible for COVID-19, SARS, and MERS epidemics have gained notable attention. In cases of COVID-19, certain individuals might develop severe complications, including pneumonia, which can be detected through lung CT scans. Studies have indicated that chest imaging plays a crucial role in the diagnosis of COVID-19 in individuals exhibiting severe symptoms. In this domain, deep learning methods, especially Convolutional Neural Networks (CNNs), have proven to be highly effective in assisting radiologists in performing various image analysis tasks related to the diagnosis of COVID-19^1^.

Deep learning models developed specifically for detecting COVID-19 infections have exhibited remarkable potential in identifying infected regions in CT scans and X-ray images. However, the training of these deep learning models necessitates access to ample and diverse medical datasets, which are typically collected from multiple sources. A majority of the current approaches rely on a centralized server for aggregating data from various healthcare institutions. This approach poses a challenge, as medical images often contain sensitive and confidential patient information, which is not permissible to share beyond the confines of the originating institution.

Federated learning (FL) emerges as a viable solution to this challenge by decentralizing the training process and retaining the data at its source. In a federated learning framework, distinct clients participate in the training process in a distributed manner using their local data. Specifically, each client independently trains a model utilizing its dataset and subsequently shares the model parameters with other participants. Crucially, the actual data does not leave the local premises, thereby maintaining the confidentiality of sensitive patient information. This approach facilitates collaborative learning without compromising data privacy.

FL can differ from centralized data sharing in a number of ways. While both approaches aim to optimize their learning objective, FL algorithms have to account for the fact that communication with clients takes place over unreliable networks with very limited upload speeds. So unlike the centralized setting in which computation is generally a bottleneck, in FL communication might be the bottleneck.

In this study, a framework was developed to facilitate collaboration among hospitals by employing multiple data sources for the detection of COVID-19 infections via FL. The framework’s decentralized data distribution ensures privacy, as data remains stored locally^2^.

## Background and Related works

Federated learning has demonstrated efficacy in an array of imaging modalities, including Magnetic Resonance Imaging (MRI)^3,4^, X-ray^5^, retinal imaging^5^, as well as in applications such as brain tumor segmentation^6,7^, diagnosis^8^, and treatment selection^7^. In particular, Federated Learning (FL) has proven to be a valuable tool for supporting physicians in their decision-making process regarding the treatment of COVID-19 patients. A landmark study that involved 20 institutions across five continents found that FL played a significant role in shaping patient treatment plans^9^. The study employed chest radiography images in conjunction with clinical information to determine the appropriate level of care and oxygen requirements for patients afflicted with COVID-19. It was observed that FL improved the performance of the predictive model, especially for institutions with smaller datasets, compared to using only local data for model training. Additionally, it was found that healthcare facilities with smaller datasets often had underrepresented categories due to a low number of patients in certain classes. The implementation of FL led to a notable improvement in predictions for these underrepresented patient categories.

**Figure 1:**
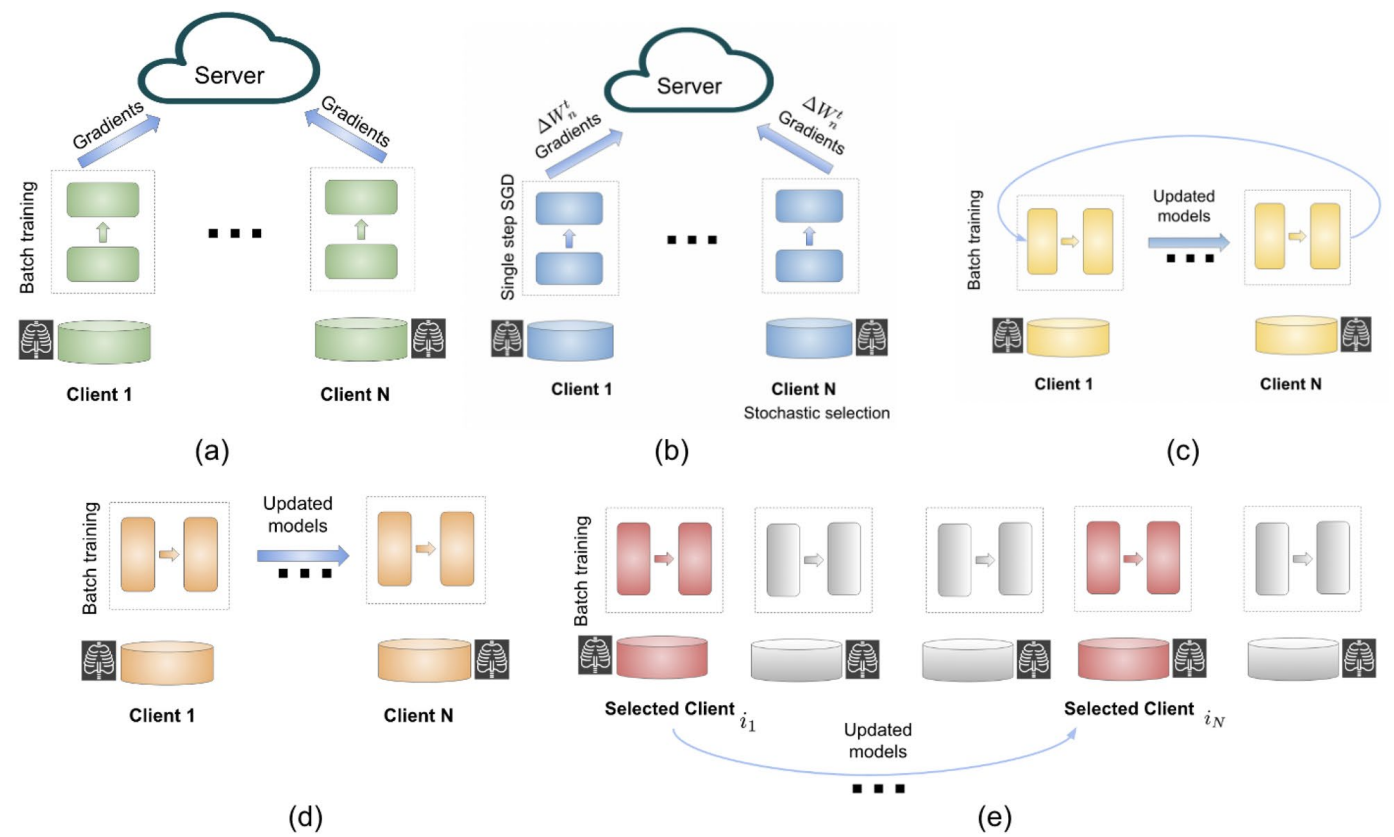
Illustration of FL models and algorithms: (**a**) Federated averaging, where clients train on a local batch of data. (**b**) FedSGD, in which a subset of clients is selected, and each performs a single step of SGD before sending model updates to the server. (**c**) Cyclic Weight Transfer (CWT), where clients train locally and pass the model to the next client, repeating the cycle. (**d**) Single Weight Transfer (SWT), where the model passes through each client only once. (**e**) Stochastic Weight Transfer (STWT), in which the model is sequentially passed through clients, with participating clients in each round being sampled randomly.

Recent research has focused on the classification of scan images to distinguish between COVID-19 patients and healthy individuals, as well as identifying lesion areas. The primary application of AI in managing COVID-19 patients has been the interpretation of radiology images, especially chest CT scans. The detection of lung alterations through these scans plays an important role in optimizing patient management and guiding treatment decisions^10–12^. Several studies have also explored 3D Convolutional neural networks^13^ and COVID-19 detection with a limited number of training samples.

While the majority of these studies report favorable accuracy, they often presume a centralized environment wherein a single data center has access to all data. However, a few studies have successfully applied distributed learning for COVID-19 detection, employing global aggregation models such as model averaging in federated learning settings^14,15^, or within a blockchain framework^16^. These studies have pointed to certain limitations of existing algorithms, such as high communication overhead^17^, as well as convergence issues or catastrophic forgetting when the number of participating hospitals increases^3,18^.

To the best of our knowledge, this is the first study to undertake a comparative analysis of multiple FL algorithms under uniform, controlled conditions in medical imaging. This comparison is crucial for evaluating the practical applicability of these algorithms. Such a comprehensive comparison is instrumental for advancing the understanding and implementation of FL in practical applications.

To evaluate the existing methods from multiple perspectives, we have implemented the most popular models and compared them in terms of performance, communication overhead, and computation burden. In the context of clinical tasks, our study is closely aligned with the current efforts in leveraging artificial intelligence for the diagnosis and management of COVID-19. The clinical relevance of our research is underscored by the significant role of chest imaging, particularly lung CT scans, in the early detection and treatment planning for COVID-19 patients. This is supported by numerous studies that highlight the effectiveness of deep learning, especially CNNs, in assisting radiologists in analyzing chest imaging for COVID-19 diagnosis. Our work extends this paradigm by employing federated learning, a method that not only leverages the advantages of deep learning in clinical imaging but also addresses critical concerns regarding data privacy and the decentralized nature of medical data.

Federated learning, in our study, is positioned as a solution to the challenges posed by traditional centralized data aggregation methods, especially in terms of privacy and data security in medical settings. The importance of our approach is further amplified considering the diverse and distributed nature of healthcare data across different institutions. The collaborative framework of federated learning, where multiple clients contribute to the training process without sharing raw data, is particularly relevant for clinical scenarios where patient confidentiality is paramount. This approach is in line with recent research that has explored distributed learning in medical imaging and its potential to improve diagnostic accuracy, particularly in facilities with limited data. Our comparison of multiple federated learning algorithms under realistic conditions aims to provide insights into their practical applicability, addressing limitations identified in previous studies such as high communication overhead and convergence challenges.

Thus, our study not only contributes to the technical field of federated learning but also holds significant implications for its clinical application in the management of COVID-19, particularly in enhancing the diagnostic process through collaborative and privacy-preserving AI models.

## Algorithms

### Centralized data sharing

In Centralized Data Sharing (CDS), data is stored in a central location and is accessible to all clients. This stands in contrast to federated and decentralized data sharing methods, where data is stored across multiple locations and accessed by either a single user or a limited number of users. CDS serves as a baseline for comparing other algorithms.

### Federated averaging

As shown in Algorithm 1, Federated Averaging involves an iterative learning procedure comprising local and global steps. In this process, each data owner trains a model received from a global server on its local dataset through local iterations^19^. Subsequently, the global server aggregates the updated local models to update the global model. This global model is then distributed to clients for the subsequent round. The optimization problem for Federated Averaging can be expressed as:

1 $$\begin{aligned} w^{t+1} = \sum \limits _{i=1}^{N}{p_{i} w_{i}^{t}}, w_{i}^{t}=\arg \min \limits _{w_{i}}{\left( {\mathcal {L}}({\mathcal {D}}_{i};w^{t})\right) } \end{aligned}$$

where *N* is the number of data owners, ${\mathcal {L}}({\mathcal {D}}_{i};w^{t})$ is a loss function indicating global model parameters $w^{t}$ of local datasets, and $p_{i}$ is the probability of selecting client *i*. Local optimization can be formulated as $w_{i}^{(t+1)} \leftarrow w^{(t)}-\eta \cdot \nabla {\mathcal {L}}(w^{(t)};{\mathcal {D}}_{i})$, where $\eta$ is the learning rate. The global model can be updated based on the local models $w_{i}$ and is shared for aggregation:

2 $$\begin{aligned} w^{(t+1)} = \sum \limits _{i=1}^{N}{p_{i} w_{i}^{(t)}} \end{aligned}$$

**Algorithm 1:**
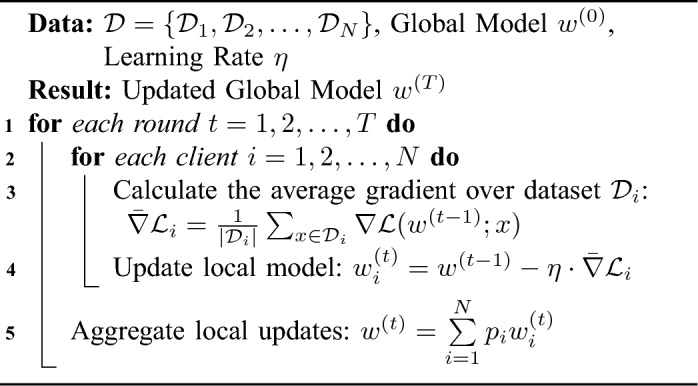
Federated averaging.

**Algorithm 2:**
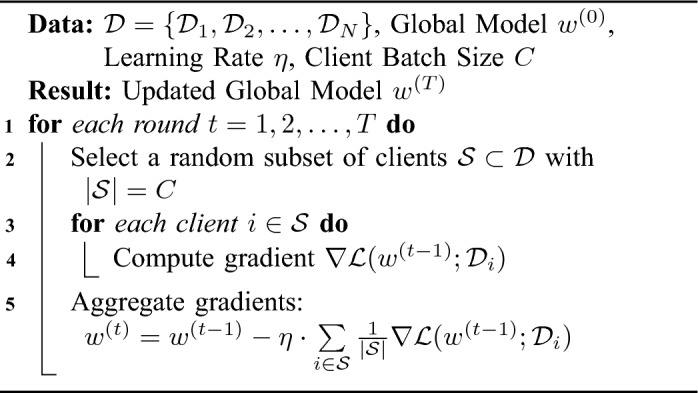
Federated stochastic gradient descent.

### Federated stochastic gradient descent

Federated Stochastic Gradient Descent, or FedSGD^20^, presented in Algorithm 2 is a variant of Federated Averaging (FedAvg) that employs a large-batch synchronous approach for multi-client learning.

From the total pool of clients, a subset is selected in each global training round, and this subset is defined by $S$. The size of $S$ is determined by the fraction $C$, which represents the proportion of the total clients to be involved in each round of training. Specifically, $|S| = C \times N$ where $N$ is the total number of clients.

In each global round, the global server dispatches the latest global model to the clients in the selected subset $S$. Each client in this subset then performs local training on their dataset for a predetermined number of epochs. After local training, the global model is updated based on the local models obtained from each client in $S$, a process similar to that in FedAvg.

However, FedSGD is distinct in its approach to gradient computation. In FedSGD, the gradient is calculated over the batch of selected clients, as defined by the subset $S$. The fraction $C$, therefore, determines the size of this batch. This approach allows for training with large batches when desired, as the gradient computation is efficiently distributed across the selected subset of clients.

The optimization problem for FedSGD can be expressed as follows:

3 $$\begin{aligned} w^{(t+1)} = w^{(t)} - \eta \cdot \sum \limits _{i=1}^{C}{p_i \cdot \nabla {\mathcal {L}} (w^{(t)};{\mathcal {D}}_i)} \end{aligned}$$

where $\eta$ is the learning rate, $p_i$ is the probability of selecting client *i* and ${\mathcal {L}}$ is the loss function. The key difference between FedAvg and FedSGD lies in the use of large-batch synchronous approach in FedSGD. This approach has been shown to outperform the naive asynchronous SGD training due to the increased accuracy and efficiency, as compared to the local training approach used in FedAvg^20,21^. Additionally, FedSGD has been shown to be more robust to non-IID data distributions, compared to FedAvg^20^.

### Cyclic weight transfer

Federated learning techniques have been widely used in medical image processing tasks using a method known as cyclic weight transfer (CWT)^5^, as shown in Algorithm 3. This method involves training models on individual clients for a number of iterations and then cyclically sharing the updated weights with the following client. However, the existing CWT algorithm faces a notable challenge, as it lacks the ability to effectively manage inter-client variability in training data or labels. To ensure the practical application of CWT, it is crucial to develop a version that can handle the common variations observed in a majority of real-world medical imaging datasets^2^.

**Algorithm 3:**
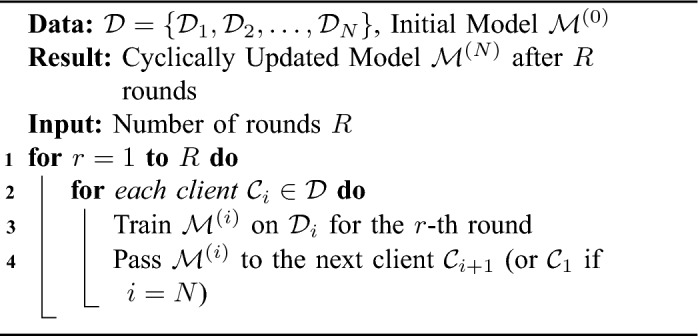
Cyclic weight transfer with stopping criteria.

**Algorithm 4:**
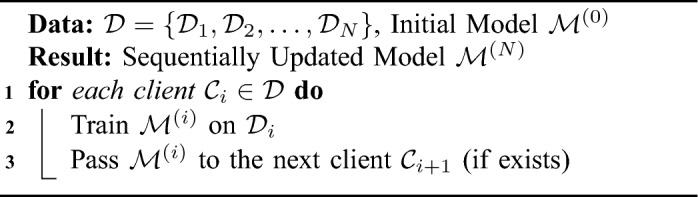
Single weight transfer.

**Algorithm 5:**
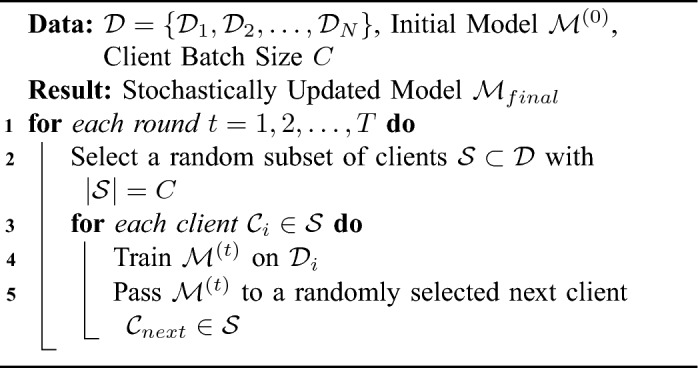
Stochastic weight transfer.

### Single weight transfer

Single weight transfer (SWT) is another FL method widely used in the medical imaging domain. In Single weight transfer, models are trained in each client with its local data, and then the updated model is transferred to the next client, as described in Algorithm 4. The difference between this method and CWT is that here the model passes each client only once^18^.

### Stochastic weight transfer

In stochastic weight transfer (STWT)^22^, we select a subsample of clients and train them in a cyclic manner. Similar to FedSGD, a ratio defines the number of selected clients to the total number of clients in each federated round, as shown in Algorithm 5.

Figure 1 provides a visual representation of the introduced algorithms. The update mechanisms of CWT, SWT, and STWT have distinct implications for the convergence and robustness of the federated learning process. CWT ensures that each client contributes to the model in a fixed order, which may lead to uniform convergence across clients. SWT avoids the potential for cyclic bias but does not guarantee that all clients will contribute equally, as the model does not revisit clients. STWT’s random client selection can provide a more robust convergence, especially in the presence of non-IID data, by preventing the model from overfitting to a particular sequence of clients. Additionally, the probability of selection $p_i$ can be incorporated into the SWT and STWT formulations to reflect the likelihood of each client being chosen for model updates, differentiating these methods from CWT.

## Experiment

### Dataset

Our experiments used two publicly available data sources, the Tongji hospital dataset^23^ and Brazil’s SARS-CoV-2 dataset^24^ Tongji dataset consists of 349 chest CT-scans of COVID-19 positive and 397 scans of healthy subjects, all low-resolution CT modalities. Brazil’s SARS-CoV-2 dataset consists of 2482 samples, 1252 scans of COVID-19-infected patients, and 1230 healthy subjects collected from multiple hospitals in Sao Paulo, Brazil. The datasets are approved by the corresponding ethical committees of each hospital, Public Hospital of Sao Paulo (HSPM), and Tongji Hospital in Wuhan, China. Train and test sets were obtained randomly from the aggregated datasets. Table 1, shows data distribution.

**Table 1:** Data distribution.

Class	Dataset	Samples	Train	Test
COVID	Brazil	1252	1451	150
	Tongji	349		
Non-COVID	Brazil	1230	1477	150
	Tongji	397		

### Preprocessing

**Figure 2:**
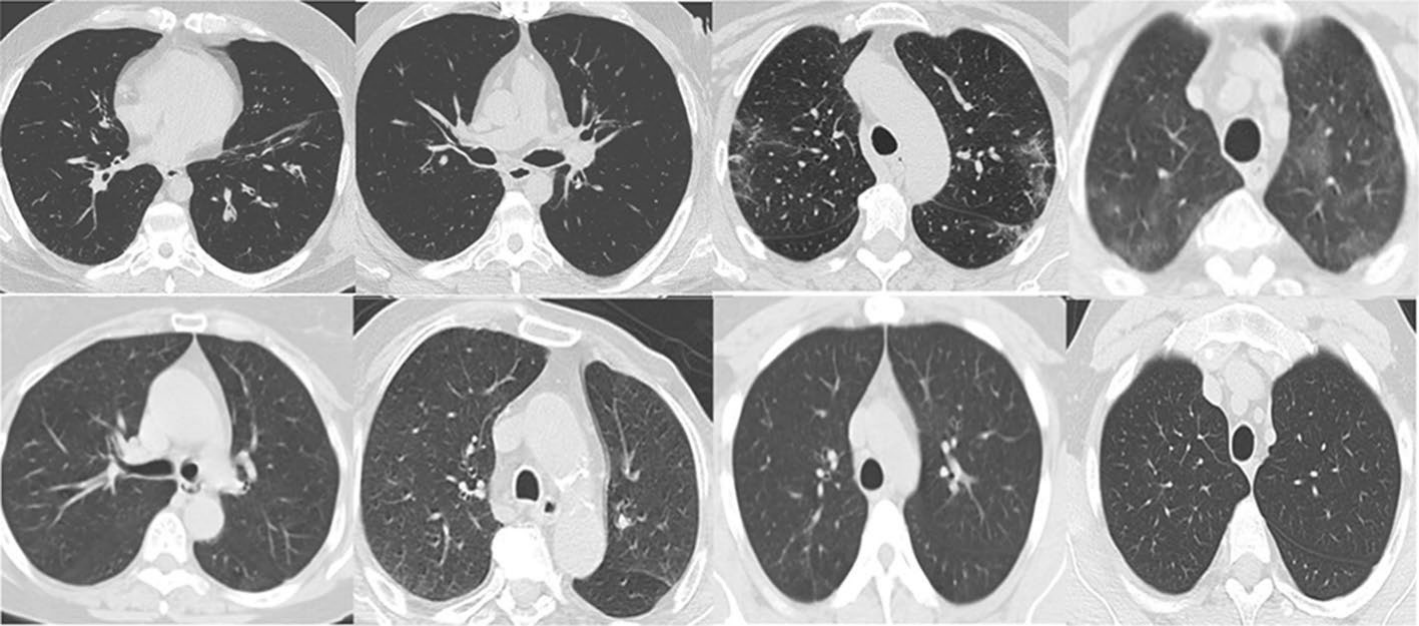
Sample CT slices of COVID-19 images (top row) and Non-COVID images (bottom row).

Images were selected as 2D slices in greyscale. Preprocessing included randomly cropping between 0.5 to full size, random horizontal flipping, and intensity normalization. CT-slices were all resized to $224 \times 224$ pixels with interpolation. Figure 2 shows samples of processed images.

### Training

**Figure 3:**
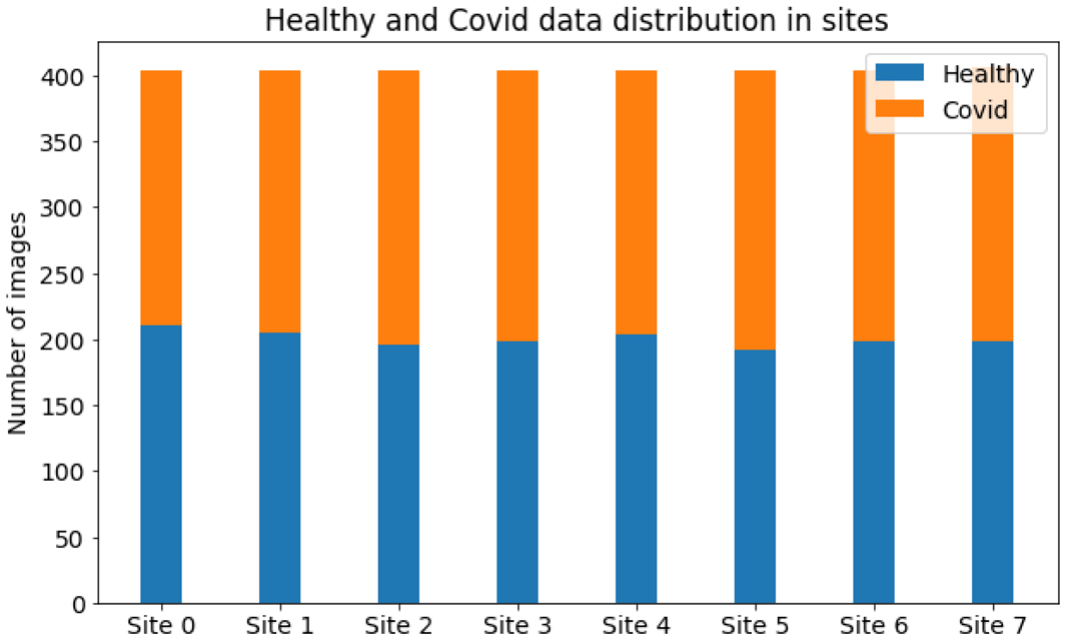
Data distribution of each client in the simulated federated setting.

ResNet-18 is used as the backbone deep learning model. ResNet-18 comprises one initial block cascaded to four middle blocks. The initial block is made of convolutional, batch normalization, ReLU, and pooling layers. Middle blocks have the same layers, connected with straight and skip connections. The model is pre-trained on ImageNet dataset^25^ with a CrossEntropy loss function and learning rate of 0.05. Each federated round consisted of 20 internal epochs for each client and batches of 16 samples in each iteration. For models which use minibatch training, like STWT and FedSGD, a subset of clients is randomly selected. Similar to training, test data was split into mini-batches, and the results were averaged across batches. We performed training with various participating clients and federated rounds to evaluate their effect on final performance. Models were also trained in a centralized, non-federated setting as well as local data training as comparison baselines. In the local training method, models were trained on data from each client individually and tested on a composite test set from other clients. For balanced testing, the 300 test samples (150 COVID-positive and 150 COVID-negative) were equally distributed among clients, ensuring each received a representative mix for evaluation. This setup aimed to emulate real-world conditions where clients train on local data but are tested on diverse external data. Figure 3 shows the data distribution among clients.

### Evaluation

Standard classification metrics, accuracy, recall, precision, and F1 score, were used as our evaluation criteria. We also evaluated the level of communication, the amount of transferred data in each algorithm, and the computational complexity of the models.

## Results

Here, the result for the setting with 10 participating clients and a maximum of 10 rounds is presented. The results are average performance among clients for all the federated rounds. Table 2 shows the results.

**Table 2:** Comparison of FL algorithms on classification of COVID-19 data for 10 clients, averaged performance in all the 10 rounds for iterative methods, with baseline methods at the bottom.

Method	Accuracy (%)	Recall (%)	Precision (%)	F1 score (%)
FedAVG	66.72	70.02	43.80	51.7
FedSGD	65.17	68.24	43.86	47.75
CWT	87.75	89.00	88.67	87.52
SWT	64.60	74.33	65.55	59.66
STWT	84.21	84.09	83.33	81.71
CDS	87.75	89.57	87.93	87.19
Local	58.18	60.40	32.38	45.77

### Assesing the impact of training rounds

To evaluate the effect of number of rounds, models with 3, 5, 10 and 15 rounds were tested. The test results are shown for both centralized and FL algorithms. Table 6 shows the results of our experiment. The increasing number of rounds correlates with higher accuracy of the global model. For sequential algorithms like CWT,SWT, STWT, defining a stopping criterion is essential for efficient and effective training. For CWT, as presented in Algorithm 3, we introduce a predetermined number of rounds, R, as the stopping criterion. This number is chosen based on empirical analysis where we monitor the loss curve to identify a point of diminishing returns. But we have also did a further examination or effect of various rounds. Typically, this is when the loss curve begins to flatten, indicating that additional rounds of training yield minimal improvements. The number of training rounds necessary to reach this point can vary depending on the dataset’s complexity, the diversity of data across clients, and the model’s architecture. In our experiments, we observed that the loss curve tends to flatten after a certain number of rounds, which we use to define our stopping criterion. However, this number might need adjustment for different datasets or federated learning configurations.

**Table 3:** Effect of number of rounds on accuracy and averaged performance metrics for FL algorithms in highly heterogeneous setting.

Method	Number of rounds and accuracy				Performance metrics (avg. every round)			
	3 rounds	5 rounds	10 rounds	15 rounds	Accuracy (%)	Recall (%)	Precision (%)	F1 score (%)
FedAVG	50.17% $\downarrow$(− 5.88%)	54.24% $\downarrow$(− 9.54%)	59.19 $\downarrow$(− 10.45%)	67.04% $\downarrow$(− 3.69%)	58.12	60.71	42.04	45.87
FedSGD	51.30% $\uparrow$(+ 0.42%)	52.29% $\downarrow$(− 3.61%)	60.59% $\downarrow$(− 14.99%)	64.25% $\downarrow$(− 12.69%)	60.09	56.97	41.44	32.18
CWT	49.95% $\downarrow$(− 30.82%)	51.30% $\downarrow$(− 38.48%)	50.84% $\downarrow$(− 40.43%)	54.39% $\downarrow$(− 39.17%)	51.75	55.06	41.10	47.62
SWT	–	–	–	–	48.60	47.42	34.78	49.30
STWT	48.38% $\downarrow$(− 42.35%)	51.65% $\downarrow$(− 32.32%)	50.64% $\downarrow$(− 38.80%)	49.01% $\downarrow$(− 43.99%)	50.91	54.02	38.64	44.71
CDS	85.06% (± 0.00%)	81.56% (± 0.00%)	91.06% (± 0.00%)	91.04% (± 0.00%)	87.75	89.57	87.93	87.19
Local	43.89% $\downarrow$(− 3.26%)	40.75% $\downarrow$(− 11.43%)	48.98% $\downarrow$(− 11.33%)	47.12% $\downarrow$(− 15.68%)	46.62	50.01	29.64	39.43

**Table 4:** Computation time for FL algorithms across varying numbers of clients.

Method	3 clients (s)	5 clients (s)	8 clients (s)	10 clients (s)
FedAVG	8934	8975	9002	9030
FedSGD	8810	8853	9013	9052
CWT	5119	5450	5383	5556
STWT	2805	5243	6101	6129
SWT	543	547	589	618

**Table 5:** Comparison of total transferred data (GB).

Method	3 rounds	5 rounds	10 rounds	15 rounds
FedAVG	1.371	2.286	4.571	6.857
FedSGD	0.823	1.371	2.743	4.114
CWT	0.686	1.143	2.286	3.428
STWT	0.411	0.686	1.371	2.057

**Table 6:** Effect number of rounds on accuracy of FL algorithms for 10 clients, 20 internal epochs.

Method	3 rounds (%)	5 rounds (%)	10 rounds (%)	15 rounds (%)
FedAVG	56.05	63.78	69.64	70.73
FedSGD	50.88	55.9	75.59	76.94
CWT	80.77	89.78	91.27	93.56
STWT	90.73	83.97	89.44	93.01
CDS	85.06	81.56	91.06	91.04
Local	47.15	52.18	60.31	62.80

### Assessing the influence of client participation

**Figure 4:**
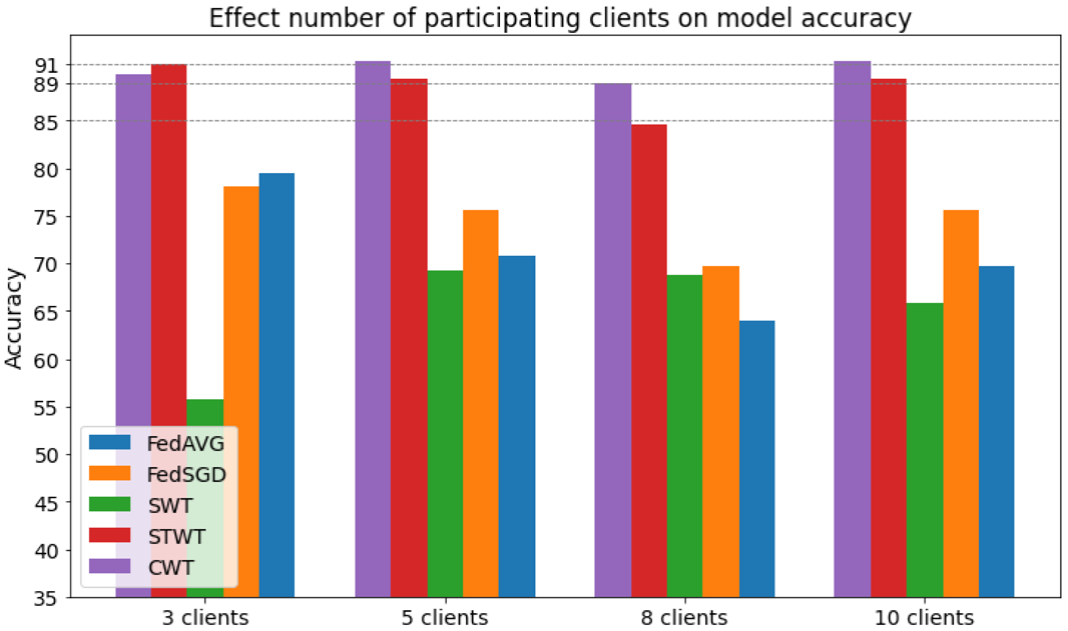
Accuracy of FL algorithms with different number of clients.

To evaluate the number of clients on the FL network, we examined scenarios with 3,5, and 8 participating clients. We trained each of the clients in 20 internal epochs. The number of Federated rounds for all the algorithms (except SWT) was 10. The average test results are shown in the Fig. 4.

### Assessing the effect of data imbalance

In our study, we aimed to evaluate the performance of federated learning models under non-IID conditions. Specifically, we assigned one data class per client to four clients: one with COVID-positive and another with COVID-negative samples from the Tongji dataset, and two more with COVID-positive and negative samples respectively from the Brazil dataset. This configuration inevitably led to biases in the clients towards the class of samples they were trained on. Our experiment was consistent with previous tests in terms of parameters: 20 internal epochs, and multiple training rounds which are detailed in Table 3. We observed significant variability in precision and recall among individual clients, with some showing values as high as 91.2% and others as low as 10.32%. This variation was dependent on whether the clients received COVID-positive or Non-COVID samples.

Our observations indicate a decrease in accuracy ranging from 5 to 15% for FedAVG and FedSGD methods under these conditions. The performance of other methods closely resembled that of a random classifier, a result that was expected given the highly heterogeneous data setup. Sequential models, in particular, exhibited challenges in this environment. These findings suggest that in cases of extreme label imbalance, such as one or two clients having exclusively one class of data, methods like FedAVG and FedSGD are more aptly suited.

Communication can also be a bottleneck in this setting. In methods like federated averaging, the lower bounds for total communicated data are proportional to $\sim$ 2*NT* where the total rounds are represented by T and the count of involved clients is denoted by N. In CWT, this lower bound is $\sim$ *NT*. In our setting, we use a ResNet 101 model. We calculated the overall transferred data for the different number of rounds. As expected, the experiments show that when clients are selected randomly, the communication time tends to be shorter compared to scenarios involving participation from all clients. Moreover, our analysis of computational costs indicates that models that do not rely on sequential processing generally demand higher computational resources compared to their sequential counterparts. The computation results are presented in Table 4, which shows the results based on the running time for a maximum of 5 federated rounds for iterative methods. The detailed results for computational and communication evaluations can be found in Tables 4 and 5, respectively.

## Discussion

Our results show that FL has comparable performance to centralized data sharing, with the advantage of keeping data private. With large volumes of data and after high number of rounds, centralized data sharing and cyclic weight transfer have the highest accuracy.

Sequential models are susceptible to catastrophic forgetting, where a global model performs well on the latest client it has seen while having poor performance in other clients. Conversely, in algorithms like FedAvg and FedSGD, the models are averaged asynchronously after all the clients have finished their training. So the trajectory is smoother and overall improving with more communication rounds. Local test results can have a high variance when passing through clients sequentially, indicating the catastrophic forgetting effect.

Models like FedAvg, and FedSGD, in which all the clients have identical copies of one global model, are slower and more challenging to converge compared to sequential models like CWT and STWT. Also, FedAvG and FedSGD require more training resources due to active server participation, resulting in more computation and network consumption. Stochastic client selection is an efficient way of training. Stochastic models save significant time and resources while having similar performance to full client participation. Overall, CWT and STWT have best results in terms of model accuracy and computation times. These findings could be practical in further federated deployments in medical institutions.

Sequential models like CWT and STWT perform better than non-sequential models on fewer training rounds. For example, after three rounds of training, STWT and CWT both reach 96% accuracy, while FedAvG reaches 66%, and FedSGD performs equally to a random classifier. As the training proceeds, FedAVG and FedSGD gradually improve with more global rounds.The concept of sequential models is similar to fine-tuning^26^ in centralized deep learning, so in cases where a hospital temporarily joins an FL network, or there is an urgency in training, sequential models are a better option.

When passing the model to the next client in CWT, ensuring the stability of training is crucial. One strategy is to implement a learning rate schedule that decreases as the number of rounds increases. This approach helps in stabilizing the learning process, especially in the later stages of training. Additionally, employing techniques like client-wise normalization or standardization of data can mitigate the impact of inter-client variability. This is particularly important in medical image processing tasks where data heterogeneity is a significant challenge.

In contrast, SWT, involves a single pass of the model through each client. This approach reduces the likelihood of cyclic bias but does not ensure equal contribution from all clients. The strategy here focuses more on efficient utilization of diverse data without revisiting clients, which can be beneficial in scenarios where data distribution is relatively uniform across clients.

STWT, adds another dimension by randomly selecting a subset of clients in each round. This method is advantageous in handling non-IID data as it prevents the model from overfitting to a specific client sequence. By incorporating a probability of selection for each client, STWT can adaptively focus on clients that provide the most informative updates, thus enhancing the robustness and convergence of the learning process.

More training rounds do not always lead to a better global model. Although average performance on all clients improves, more global rounds lead to worse performance for some clients. The global model can overfit some clients, leading to lower performance on others^27^. Some studies suggested early stopping and fine-tuning to local dataset after global training is finished^28^. In all the algorithms, more clients resulted in slower convergence. This effect is stronger in the FedAvg algorithm. In FedAVG, the Global model must compromise between potentially disparate local minima^29^. Methods such as adaptive or stochastic selection of clients and momentum-based models help faster convergence^30^. Our results suggest that stochastic client participation is close to full client participation. The average results of four trials with varying rounds, shown in Table 6 indicate that stochastic client participation in FedSGD results in 5.23% performance loss and 40% less bandwidth consumption compared to FedAvg. In STWT, it results in only 1.25% less accuracy but saves 40% of communication and 11.3% of computation. These results are in accordance with prior studies, showing that, in theory, stochastic and full client participation have similar global minima^31^. Stochastic client selection can be advantageous when there are limited resources, or in larger networks with occasionally unavailable clients.

We recommend the non-sequential approaches, such as FedAVG, FedSGD and their variants as effective federated learning methods for heterogeneous COVID-19 detection tasks. This recommendation is based on several key observations. FedAVG has demonstrated high resilience to the non-IID nature of our datasets. Despite the inherent data variability and imbalances, FedAVG consistently showed more reliable performance across different clients. Also, Given the extensive size of the datasets, particularly the Brazil dataset, FedAVG’s ability to efficiently handle large volumes of data while maintaining computational feasibility stands out. Our experiments indicate that FedAVG, while simplistic in its approach, offers a strong balance between accuracy and generalizability.

We did not assume any shift in clients’ data. A more comprehensive analysis should consider the effect of the domain and distribution shifts on the performance of the algorithms. Also, inter-client data variability and the effect of heterogenous clients could be a future line of research.

## Conclusion

FL enables extensive collaborations of hospitals to address medical imaging problems while keeping data private. Real-world implementation requires consideration of efficiency and hardware requirements in addition to model performance, especially in the healthcare field, which generally has limited infrastructure. We implemented five FL algorithms for COVID-19 detection and analyzed their efficiency and accuracy. Our results suggest that FL algorithms have comparable performance to centralized data sharing, with the advantage of keeping data private. They also show that the sequential methods are a better option in most of the scenarios. This study can be helpful in the deployment of FL systems in COVID-19 detection and medical image analysis in general. It is crucial to acknowledge the potential limitation of catastrophic forgetting, particularly in the context of federated learning with larger datasets. Catastrophic forgetting occurs when a learning model, upon being trained on new data, loses the information previously learned, as we have observed on our experiments. This challenge is particularly relevant when applying federated learning methods to extensive and diverse datasets, where the balance between learning new patterns and retaining previously acquired knowledge is critical. Our study provides foundational insights, but future research should carefully consider and address this phenomenon, especially when scaling up to larger, more complex datasets. Recognizing and mitigating catastrophic forgetting is essential for ensuring the robustness and reliability of federated learning models in practical, real-world applications.

## Data Availability

The datasets used in this study are publicly available. The Tongji hospital dataset and Brazil’s SARS-CoV-2 dataset used can be accessed on Kaggle at the following links: https://www.kaggle.com/datasets/ahmedtronic/covid-19 and https://www.kaggle.com/datasets/plameneduardo/sarscov2-ctscan-dataset.
